# Application of *Toxoplasma gondii*-specific SAG1, GRA7 and BAG1 proteins in serodiagnosis of animal toxoplasmosis

**DOI:** 10.3389/fcimb.2022.1029768

**Published:** 2022-12-15

**Authors:** Tongsheng Qi, Jingkai Ai, Yali Sun, Hejia Ma, Ming Kang, Xiaoqian You, Jixu Li

**Affiliations:** ^1^ State Key Laboratory of Plateau Ecology and Agriculture, Qinghai University, Xining, China; ^2^ College of Agriculture and Animal Husbandry, Qinghai University, Xining, China; ^3^ Qinghai Provincial Key Laboratory of Pathogen Diagnosis for Animal Diseases and Green Technical Research for Prevention and Control, Qinghai University, Xining, China; ^4^ Qinghai Animal Disease Control Center, Department of Agriculture and Rural Affairs of Qinghai Province, Xining, China

**Keywords:** *Toxoplasma gondii*, SAG1, GRA7, BAG1, IgG, IgM, animals

## Abstract

Toxoplasmosis is a zoonotic disease caused by the obligate intracellular protozoan parasite *T. gondii* which is widely prevalent in humans and animals worldwide. The diagnosis of toxoplasmosis and distinguishing acute or chronic *T. gondii* infections have utmost importance for humans and animals. The *Tg*SAG1, *Tg*GRA7, and *Tg*BAG1 proteins were used in the present study to develop the serological rSAG1-ELISA, rGRA7-ELISA and rBAG1-ELISA methods for the testing of *T. gondii* specific IgG and IgM antibodies and differentiating acute or chronic toxoplasmosis in 3733 animals, including Tibetan sheep, yaks, pigs, cows, cattle, horses, chickens, camels and donkeys from the Qinghai-Tibetan Plateau. The ELISA tests showed that the overall positivity of IgG antibody was 21.1% (786/3733), 15.3% (570/3733) and 18.2% (680/3733) for rSAG1-, rGRA7- and rBAG1-ELISA, respectively, and the positivity of IgM antibody was 11.8% (439/3733), 13.0% (486/3733) and 11.8% (442/3733) for rSAG1-, rGRA7- and rBAG1-ELISA, respectively. A total of 241 animals (6.5%) positive for all rSAG1-, rGRA7- and rBAG1-IgG were found in this study, and the 141 animals (3.8%) tested were anti-*T. gondii* IgM positive in all three ELISAs. Moreover, the 338, 284 and 377 animals were IgG positive in rSAG1 + rGRA7-, rBAG1 + rGRA7- and rSAG1 + rBAG1- ELISAs respectively, and the 346, 178 and 166 animals in rSAG1 + rGRA7-, rBAG1 + rGRA7- and rSAG1 + rBAG1-ELISAs were IgM positive respectively. The results confirmed that the application of SAG1, GRA7, and BAG1 recombinant antigens could successfully be used in the detection of specific IgG and IgM antibodies for distinguishing between acute or chronic *T. gondii* infections. It is inferred that the forms in which current animal species in the plateau area were infected with *T. gondii*, and the period of infection or the clinical manifestations of the current infections may be different. The present study provides substantial clinical evidence for the differential diagnosis of toxoplasmosis, and the classification of acute and chronic *T. gondii* infections.

## 1 Introduction

Toxoplasmosis is a zoonotic disease caused by the obligate intracellular protozoan parasite *T. gondii* which is widely prevalent among humans and animals worldwide (Robert-Gangneux and Dardé, 2012; Halonen and Weiss, 2013; Liu et al., 2015; Matta et al., 2021). Humans and animals can become the intermediate hosts of *T. gondii* through the ingestion of foods or water contaminated with oocysts of *T. gondii* shed with definitive host cats, or by eating undercooked or raw meats that contain tissue cysts of *T. gondii* (Robert-Gangneux and Dardé, 2012; Halonen and Weiss, 2013; Liu et al., 2015; Matta et al., 2021). *T. gondii* infections are characterized by significant morbidity and mortality in immunocompromised patients, such as individuals with AIDS, and serious congenital immune defects (Tenter et al., 2000). Common toxoplasmosis among both weakened humans and animals leads to neurological, ocular, and systemic diseases or abortion, stillbirths, and abnormalities or becoming carrier (Tenter et al., 2000). Thus, the infection of *T. gondii* is divided into acute infection caused by oocysts (sporozoite form) or tachyzoites and chronic infection caused by bradyzoites in a long-term presence in the host tissues (Dubey et al., 1998; Lyons et al., 2002). This chronic or latent infection generally exhibits a benign course in the immunocompetent population but can reactivate in people with weak immune systems (Montoya and Liesenfeld, 2004; Robert-Gangneux and Dardé, 2012; Saadatnia and Golkar, 2012; Lourido, 2019). Therefore, the diagnosis of toxoplasmosis and distinguishing acute or chronic *T. gondii* infections are important for humans and animals.

The diagnosis of toxoplasmosis can be achieved using microscopic examination, *in vitro* culture, animal inoculation, and molecular and serological methods, while the common approach is the serological assays using *T. gondii* tachyzoite lysate antigen or specific antigens (Terkawi et al., 2013; Döşkaya et al., 2014). ELISAs are reliable serological tests that have been developed for the detection of *T. gondii* infection in animals, and the key component of these methods is the selection of antigens with strong specificity and high sensitivity (Terkawi et al., 2013; Döşkaya et al., 2014). Recently, ELISA methods based on recombinant proteins have been developed to diagnose acute toxoplasmosis, such as surface-related proteins (SRS family), dense granule proteins (GRAs), and rhoptry proteins (ROPs) (Pietkiewicz et al., 2004; Terkawi et al., 2013; Döşkaya et al., 2014; Holec-Gąsior et al., 2014; Xicoténcatl-García et al., 2019; Teimouri et al., 2021). Among these *T. gondii* specific antigens, *Tg*SAG1 and *Tg*GRA7 are highlighted and have been widely used serologically to diagnose *T. gondii* infection (Terkawi et al., 2013; Xicoténcatl-García et al., 2019; Teimouri et al., 2021). *Tg*SAG1 is expressed on the surface of tachyzoites, which is a highly antigenic protein widely used for the diagnosis of *T. gondii* infection (Holec-Gąsior et al., 2014; Xicoténcatl-García et al., 2019; Teimouri et al., 2021). *Tg*GRA7 is a secreted protein and expressed by sporozoites, tachyzoites and early-stage bradyzoites, and GRA7 produces a very strong antibody response in the acute phase of infection caused by all three parasite forms (Terkawi et al., 2013; Xicoténcatl-García et al., 2019; Teimouri et al., 2021). Moreover, *Tg*BAG1 which is only expressed in bradyzoites, is a specific and characterized protein of the bradyzoite form and is a marker of *T. gondii* cyst infections (Döşkaya et al., 2014). The specific *T. gondii* antigens to sporozoite, tachyzoite and bradyzoite forms could be used to predict the infection stage.

Distinguishing between acute and chronic *T. gondii* infections could be achieved based on serological detection of immunoglobulin M (IgM) and immunoglobulin G (IgG) data (Montoya and Remington, 2008; Dhakal et al., 2015). Hence, the current study utilized the *Tg*SAG1, *Tg*GRA7, and *Tg*BAG1 proteins to develop serological rSAG1-, rGRA7- and rBAG1-ELISAs to test *T. gondii* specific IgG and IgM antibodies for distinguishing acute or chronic *T. gondii* infections in Tibetan sheep (*Ovis aries*), yaks (*Bos grunniens*), pigs (*Sus domesticus*), cows (*Bos taurus*), cattle (*Bos taurus domestica*), horses (*Equus ferus caballus*), chickens (*Gallus gallus domesticus*), camels (*Camelus bactrianus*) and donkeys (*Equus asinus*) from the Qinghai-Tibetan Plateau area.

## 2 Materials and methods

### 2.1 Serum collection from various animals in the Qinghai-Tibetan Plateau

In this study, a total of 3733 animal blood samples were collected from Tibetan sheep, yaks, pigs, cows, cattle, horses, chickens, camels and donkeys at 49 sampling sites in the 5 states and 2 cities in the Qinghai-Tibetan Plateau area (QTPA) with geographical coordinates of 31°36′-39°19′ N and 89°35′-103°04′ E from June 2021 to February 2022 as shown in **Figure S1** and **Table S1**. Centrifugation of the fresh blood from different animals was performed at 5000 rpm for 10 minutes. The serum from the supernatant was transferred to new collection tubes. After excluding the unqualified serum (such as hemolytic samples), animal serum samples were frozen and stored at -80°C until assayed. All procedures were carried out according to the ethical guidelines of Qinghai University.

### 2.2 Cloning and expression of *T. gondii* specific-SAG1, GRA7 and BAG1 proteins

Amino acid sequence alignment and phylogenetic analyses for *Tg*SAG1, *Tg*GRA7, and *Tg*BAG1 with the related cycle-forming organizations (*Neospora caninum*, *Besnoitia species*, *Sarcocystis* species, etc.) were constructed using the maximum lifestyle statistical method and bootstrap analysis with 500 replications in MEGA7, and the sequences included *T. gondii* SAG1 (AFO54849.1), *N. caninum* SAG1 (AAD25091.1), *Sarcocystis neurona* SAG1 (AAK40366.1), *T. gondii* GRA7 (ABE69193.1), *N. caninum* GRA7 (AFB77190.1), *Besnoitia besnoiti* GRA7 (XP_029218567.1), *T. gondii* BAG1 (XP_002365116.1), *N. caninum* BAG1 (BAI44436.1), and *B. besnoiti* BAG1 (XP_029221932.1). The pGEX-4T-3-SAG1 and pGEX-4T-3-GRA7 plasmids from previous studies were used to produce recombinant *Tg*SAG1-GST and *Tg*GRA7-GST proteins (Kimbita et al., 2001; Masatani et al., 2013). The *BAG1* gene (*Toxoplasma* Genomics Resource TGME49_259020) was amplified by PCR from the cDNA of *T. gondii*. The primers that included a BamHI site (underlined) in the forward primer 5’- CGC GGATCC ATG GCG CCG TCA GCA TCG CAT -3’ and a NotI site (underlined) in the reverse primer 5’- ATAAGAAT GCGGCCGC CTA CTT CAC GCT GAT TTG TTG CT-3’ for the *BAG1* gene were used. The PCR products were digested with BamH I and Not I, and inserted into the pGEX-4T-1 plasmid vector treated with the same restriction enzymes (Roche, Switzerland). The recombinant pGEX-4T-1 empty vector, pGEX-4T-3-SAG1, pGEX-4T-3-GRA7, and pGEX-4T-1-BAG1 were expressed as glutathione s-transferase (GST) fusion proteins in *Escherichia coli* BL21(DE3) (New England BioLabs, USA) and purified with Glutathione-Sepharose 4B beads (GE Healthcare Life Sciences, USA) according to the manufacturer’s instructions. The final concentrations of GST, r*Tg*SAG1-GST, r*Tg*GRA7-GST, and r*Tg*BAG1-GST proteins were measured with a bicinchoninic acid protein assay kit (Thermo Fisher Scientific, USA) before being used.

### 2.3 Development of indirect ELISAs based on rSAG1, rGRA7 and rBAG1 proteins

In this study, the r*Tg*SAG1-GST, r*Tg*GRA7-GST, and r*Tg*BAG1-GST proteins were used to establish the indirect rSAG1-ELISA, rGRA7-ELISA and rBAG1-ELISA to detect both IgG and IgM antibodies against *T. gondii* in the 3733 animals, including the 904 in Tibetan sheep, 752 in yaks, 496 in cows, 456 in pigs, 451 in cattle, 389 in horses, 199 in chickens, 49 in camels and 37 in donkeys. Briefly, the 1 μg/mL recombinant proteins were diluted in coating buffer (0.05 M carbonate-bicarbonate, pH 9.6). The animal sera were diluted by 1:100, and the secondary antibodies of anti-bovine, cow, sheep, horse, pig, chicken, donkey, and camel IgG or IgM as listed in **Table S2**, were diluted 1:3000-4000. The ABTS, (2,2’-azino-bis(3-ethylbenzothiazoline-6-sulfonic acid)) substrate, was used to show the OD 415 nm values. The soluble GST protein was used as the control under consistent experimental conditions with three ELISAs. In this study, the 5 positive and negative mouse sera for anti-*T. gondii*, *Neospora caninum* and *Sarcocystis gigantea* were used to assess the specificity of IgG-ELISA and IgM-ELISA based on GRA7, SAG1, BAG1, and GST proteins, respectively. Moreover, the coated *Tg*GRA7, *Tg*SAG1, *Tg*BAG1, and GST proteins at 2, 1, 0.5, 0.25, 0.125, and 0.0625 μg/mL, or anti-*T. gondii* positive and negative sera diluted 1:100, 200, 400, 800, 1600, 3200, 6400, 12800 and 25600, were used to develop the IgG-ELISAs and IgM-ELISAs for analyzing the sensitivity of the current tests, respectively. Moreover, the positive and negative animal serum samples for *T. gondii* IgG and IgM antibodies confirmed by the commercial ID Screen^®^ Toxoplasmosis Indirect Multi-species ELISA kit (ID.vet, France) and our previous study (Li et al., 2021) were used as controls. All controls were re-confirmed using the ELISA tests based on tachyzoite and bradyzoite lysate antigens under the current protocol. Furthermore, the commercial kit (ID Screen^®^ Toxoplasmosis Indirect Multi-species ELISA, ID.vet, Grabels, France) was used to confirm the currently determined seropositive and seronegative samples from at least one animal species.

### 2.4 Data analysis

For the resulting judgment, the cut-off points were calculated as the mean values of OD 415 nm for the negative sera (including the serum samples of 30 Tibetan sheep, 30 yaks, 30 cows, 30 pigs, 30 cattle, 20 horses, 20 chickens, 5 camels, and 5 donkeys) kept in our lab (Qinghai University, Xining, China) (Li et al., 2021) plus three times the standard deviations of OD 415 nm values of these negative controls: the mean (X) and standard deviation (SD) of the negative control results were calculated, and the X+3SD was the cut-off values. The OD 415 values of the tested animals were greater than the respective cut-off values assessed as positive. For the weak positive samples with similar cut-off values or lighter colors were repeated with the second doubtful result being treated as negative samples. To graph and analyze the data, GraphPad Prism 8 software (GraphPad Software Inc., USA) was used. The prevalence and 95% confidence intervals per pathogen species were calculated using the OpenEpi program (http://www.openepi.com/Proportion/Proportion.htm). The chi-squared test was used to compare the proportions of determined seropositivity in animals from different regions. The differences were considered to be statistically significant when the resulting *P*-values were < 0.05.

## 3 Results

### 3.1 Establishment of rSAG1-ELISA, rGRA7-ELISA and rBAG1-ELISA tests

**Figure 1:**
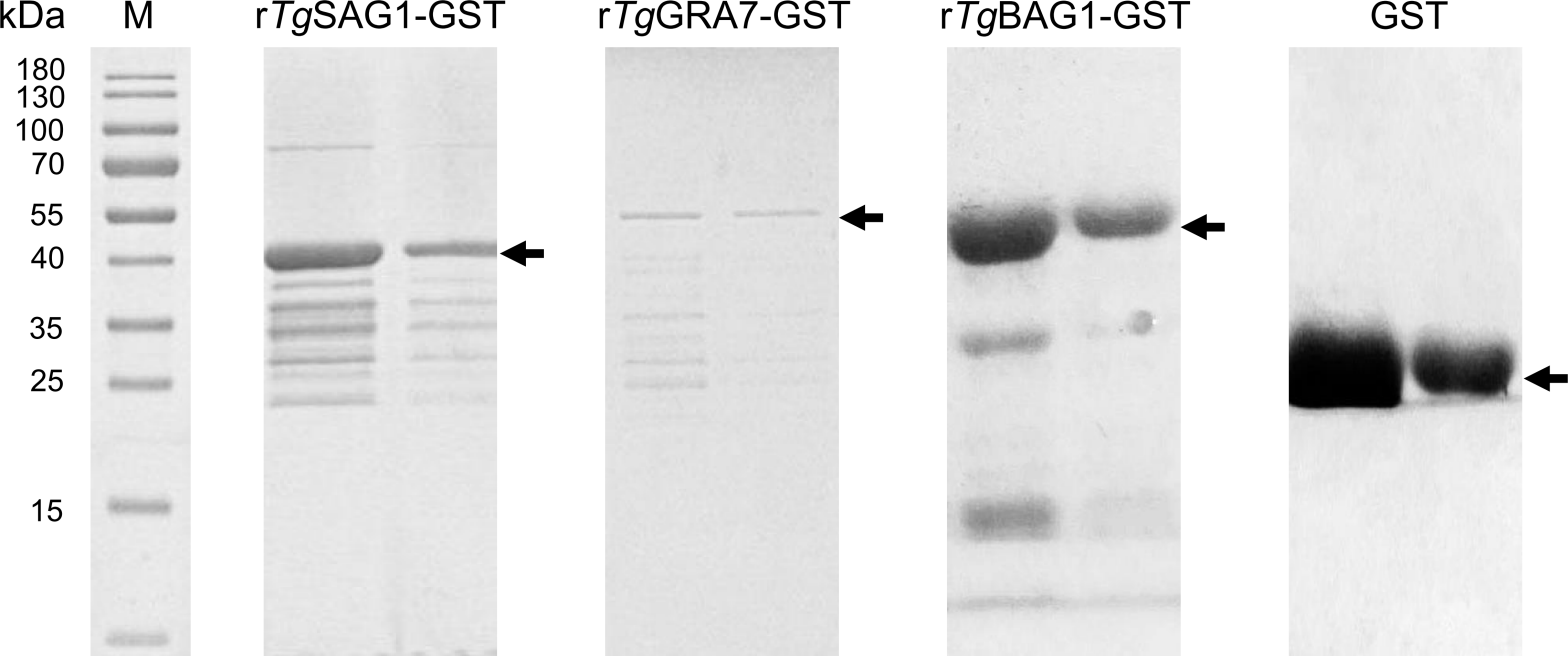
The recombinant SAG1, GRA7 and BAG1 proteins of *T. gondii* was expressed. SDS-PAGE analysis showed the rSAG1, rGRA7, rBAG1 and GST proteins.

In this study, the amino acid sequence alignment analysis was performed on the SAG1, GRA7, and BAG1 proteins of *T. gondii* with the related cyst-forming organisms (*Neospora caninum*, *Besnoitia* species, *Sarcocystis* species, etc.), and shared the homology of 5.0-86.8% (**Figure S2** **).** The r*Tg*SAG1-GST, r*Tg*GRA7-GST, and r*Tg*BAG1-GST proteins were expressed (**Figure 1**), and used to establish the indirect rSAG1-ELISA, rGRA7-ELISA and rBAG1-ELISA methods for identifying the carrier animals of *T. gondii* specific-IgG and IgM antibodies, and comparing the differences among three antigens in the serodiagnosis of animal toxoplasmosis in various animals from the Qinghai-Tibetan Plateau.

The IgG-ELISAs (**Figure S3A**) and IgM-ELISAs (**Figure S3B**) based on *Tg*GRA7, *Tg*SAG1, *Tg*BAG1, and GST proteins were performed to detect mouse anti-*T. gondii*, *N. caninum*, and *S. gigantea* positive sera to analyze the specificity of the current ELISA tests in this study respectively. The results showed that GRA7-, SAG1-, and BAG1-ELISAs only reacted with the positive sera of *T. gondii* but did not respond to the positive sera of *N. caninum* and *S. gigantea* (**Figure S3**). The GST protein did not react with any serum (**Figure S3**). In addition, the results show that the current ELISA reactions could occur when the concentration of coated proteins was very low under a positive serum dilution of 1:100 (**Figure S4**). Moreover, the OD values at the serum dilution of 1:800-6400 were still greater than the cut-off values (calculated using the OD values of negative controls), while GST protein did not demonstrate any response (**Figure S5**). These results suggest that the detection methods employed were highly specific and sensitive.

**Figure 2:**
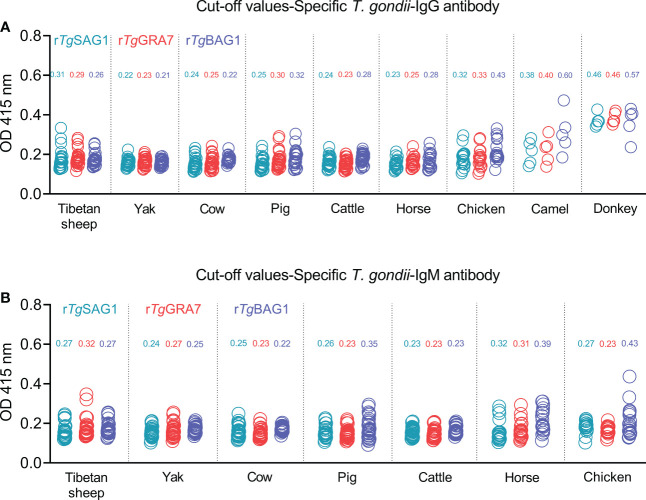
Establishment of rSAG1-ELISA, rGRA7-ELISA and rBAG1-ELISA tests and calculating the cut-off values for *T. gondii* IgG **(A)** and IgM **(B)** antibodies.

The 5-30 negative sera confirmed by our previous study (Li et al., 2021) and the commercial kits were used to detect OD 415 values in the different animal species, and calculate the cut-off values for IgG and IgM determination in Tibetan sheep, yaks, cows, pigs, cattle, horses, chickens, camels, and donkeys as shown in **Figures 2A, B**. The glutathione s-transferase (GST)-ELISA tests showed no-reaction with *T. gondii* positive sera under the same experimental conditions (**Figure S3**).

### 3.2 Detection of *T. gondii* IgG and IgM antibodies in animals

**Figure 3:**
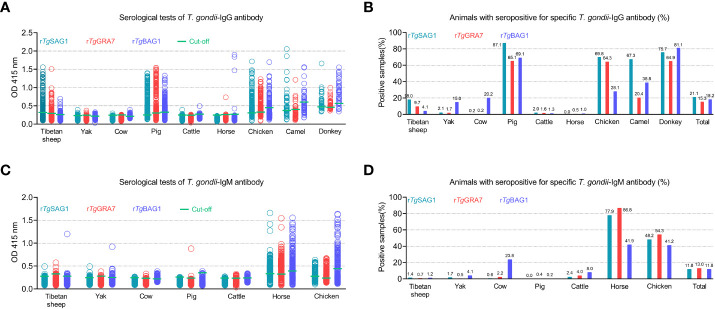
Detection of *T. gondii* IgG **(A, B)** and IgM **(C, D)** antibodies in various animals in the Qinghai-Tibet Plateau by the indirect ELISA methods based on SAG1, GRA7 and BAG1 antigens in this study.

As shown in **Figure 3** and **Table 1**, rSAG1-ELISA, rGRA7-ELISA and rBAG1-ELISA were used to detect *T. gondii* IgG and IgM antibodies in the same animals. The results showed that the overall positivity of IgG antibody was 21.1% (786/3733), 15.3% (570/3733) and 18.2% (680/3733) for rSAG1-ELISA, rGRA7-ELISA and rBAG1-ELISA respectively (**Figures 3A, B**), and the 11.8% (439/3733), 13.0% (486/3733) and 11.8% (442/3733) of the animals were IgM antibody-positive for rSAG1-ELISA, rGRA7-ELISA and rBAG1-ELISA respectively (**Figures 3C, D**).

**Table 1:** Seroprevalence of specific-*T. gondii* IgG and IgM antibodies in various animals.

Animal	No. tested	IgG-SAG1 (%, 95% CI)	IgG-GRA7 (%, 95% CI)	IgG-BAG1 (%, 95% CI)	IgM-SAG1 (%, 95% CI)	IgM-GRA7 (%, 95% CI)	IgM-BAG1 (%, 95% CI)
Tibetan sheep	904	163 (18.0, 15.5-20.5)	88 (9.7, 7.8-11.7)	37 (4.1, 2.8-5.4)	13 (1.4, 0.7-2.2)	6 (0.7, 0.1-1.2)	11 (1.2, 0.5-1.9)
Yak	752	16 (2.1, 1.1-3.2)	13 (1.7, 0.8-2.7)	113 (15.0, 12.5-17.6)	13 (1.7, 0.8-2.7)	4 (0.5, 0.0-1.1)	31 (4.1, 2.7-5.5)
Cow	496	1 (0.2, 0.2-0.6)	1 (0.2, 0.2-0.6)	100 (20.2, 16.6-23.7)	3 (0.6, 0.1-1.3)	11 (2.2, 0.9-3.5)	118 (23.8, 20.0-27.5)
Pig	456	397 (87.1, 84.0-90.1)	297 (65.1, 60.8-69.5)	315 (69.1, 64.8-73.3)	0	2 (0.4, 0.2-1.0)	1 (0.2, 0.2-0.6)
Cattle	451	9 (2.0. 0.7-3.3)	7 (1.6, 0.4-2.7)	6 (1.3, 0.3-2.4)	11 (2.4, 1.0-3.9)	18 (4.0, 2.2-5.8)	36 (8.0, 5.5-10.5)
Horse	389	0	2 (0.5, 0.2-1.2)	4 (1.0, 0.0-2.0)	303 (77.9, 73.8-82.0)	337 (86.8, 83.3-90.0)	163 (41.9, 37.0-46.8)
Chicken	199	139 (69.8, 63.5-76.2)	128 (64.3, 57.7-71.0)	56 (28.1, 21.9-34.4)	96 (48.2, 41.3-55.2)	108 (54.3, 47.3-61.2)	82 (41.2, 34.4-48.0)
Camel	49	33 (67.3, 54.2-80.5)	10 (20.4, 9.1-31.7)	19 (38.8, 25.1-52.4)	/	/	/
Donkey	37	28 (75.7, 61.9-89.5)	24 (64.9, 49.5-80.2)	30 (81.1, 68.5-93.7)	/	/	/
Total	3733	786 (21.1, 19.7-22.4)	570 (15.3, 14.1-16.4)	680 (18.2, 17.0-19.5)	439 (11.8, 10.7-12.8)	486 (13.0, 11.9-14.1)	442 (11.8, 10.8-12.9)

%, Prevalence. 95% CI, 95% Confidence Interval./, no tested.

In ELISA tests, the pig was the most prevalent animal in both rSAG1-ELISA and rGRA7-ELISA and the donkey was the most prevalent animal in rBAG1-ELISA for IgG positivity, while horses had the highest IgM positivity according to all three methods (**Figure 3** and **Table 1**). Specifically, our results revealed that the animals with the next highest IgG positivity rates in the rSAG1-tests were donkeys, chickens, camels, Tibetan sheep, yaks, cattle and cows; those with the next highest rates in the rGRA7- tests were donkeys, chickens, camels, Tibetan sheep, yaks, cattle, horses and cows; and those with the next highest rates in the rBAG1-IgG tests were pigs, camels, chickens, cows, yaks, Tibetan sheep, cattle and horses. However, the results showed that the animals with the next highest positivity rates of IgM antibodies in the rSAG1- tests were followed by chickens, cattle, yaks, Tibetan sheep and cows; those in the rGRA7- tests were followed by chickens, cattle, cows, Tibetan sheep, yaks and pigs; those in the rBAG1-IgG tests were followed by chickens, cows, cattle, yaks, Tibetan sheep and pigs. Moreover, the results of the analysis of the positivity of animals for both IgG and IgM antibodies based on the same antigen, revealed that 2.0%, 2.5% and 1.5% of the animals exhibited both IgG and IgM positivity in the rSAG1-ELISA, rGRA7-ELISA and rBAG1-ELISA tests, respectively (**Table 2**).

**Table 2:** Animals with seropositive for both IgG and IgM antibodies in rSAG1-ELISA, rGRA7-ELISA and rBAG1-ELISA tests.

Animal	No. tested	rSAG1-ELISA (%, 95% CI)	rGRA7-ELISA (%, 95% CI)	rBAG1-ELISA (%, 95% CI)
Tibetan sheep	904	3 (0.3, 0.0-0.7)	4 (0.4, 0.0-0.9)	2 (0.2, 0.1-0.5)
Yak	752	0	0	7 (0.9, 0.2-1.6)
Cow	496	0	0	44 (8.9, 6.4-11.4)
Pig	456	0	2 (0.4, 0.2-1.0)	0
Cattle	451	0	2 (0.4, 0.2-1.1)	0
Horse	389	0	1 (0.3, 0.2-0.8)	1 (0.3, 0.2-0.8)
Chicken	199	71 (35.7, 29.0-42.3)	83 (41.7, 34.9-48.6)	3 (1.5, 0.2-3.2)
Camel	49	/	/	/
Donkey	37	/	/	/
Total	3733	74 (2.0, 1.5-2.4)	92 (2.5, 2.0-3.0)	57 (1.5, 1.1-1.9)

%, Prevalence. 95% CI, 95% Confidence Interval./, no tested.

In order to confirm the current ELISAs, the 50 seropositive and 50 seronegative samples determined with the current IgG-ELISA based on SAG1 in pigs were selected to compare with the commercial kits based on *Tg*SAG1 protein. The results showed an 82% match rate between the kits and the current SAG1-IgG ELISA, including a 68% match rate for positive and 92% for negative samples (**Table S3**). According to these results (**Table S3**), we calculated that the serological *T. gondii* IgG-ELISA assay based on the SAG1 protein established in pigs in this study had a sensitivity of 89.7% and a specificity of 75.4%.

### 3.3 Analysis of animal co-positive for rSAG1-ELISA, rGRA7-ELISA and rBAG1-ELISA

A total of 241 animals (6.5%) with all rSAG1-, rGRA7- and rBAG1-IgG positivity were found in this study (**Table 3**), and the 141 animals (3.8%) tested which included 130 horses and 11 chickens were anti-*T. gondii* IgM positive in all three ELISAs (**Table 4**). Moreover, a total of 338, 284 and 377 animals were IgG positive in the rSAG1 + rGRA7-, rBAG1 + rGRA7- and rSAG1 + rBAG1- ELISAs respectively (**Table 3**), and 346, 178 and 166 animals in the rSAG1 + rGRA7-, rBAG1 + rGRA7- and rSAG1 + rBAG1-ELISAs were IgM positive respectively (**Table 4**). Furthermore, the 1796 animals (48.1%) were positive animals for IgG and IgM antibodies in at least one specific *T. gondii* antigen, and 29.8% of animals (1111/3733) were IgG-positive for at least one specific *T. gondii* antigen and a total of 825 animals (22.1%) were IgM-positive for at least one antigen (**Table 5**).

**Table 3:** Specific-*T. gondii* IgG-positive animals based on among SAG1, GRA7 and BAG1 proteins.

Animal	No. tested	Both IgG-SAG1 and IgG-GRA7 positive (%, 95% CI)	Both IgG-BAG1 and IgG-GRA7 positive (%, 95% CI)	Both IgG-SAG1 and IgG-BAG1 positive (%, 95% CI)	Among IgG-SAG1, -GRA7 and-BAG1 positive (%, 95% CI)
Tibetan sheep	904	28 (3.1, 2.0-4.2)	5 (0.6, 0.1-1.0)	10 (1.1, 0.4-1.8)	2 (0.2, 0.1-0.5)
Yak	752	0	1 (0.1, 0.1-0.4)	3 (0.4, 0.1-0.8)	0
Cow	496	0	0	0	0
Pig	456	187 (41.0, 36.5-45.5)	208 (45.6, 41.0-50.2)	279 (61.2, 56.7-65.7)	182 (39.9, 35.4-44.4)
Cattle	451	0	0	1 (0.2, 0.2-0.7)	0
Horse	389	0	0	0	0
Chicken	199	98 (49.2, 42.3-56.2)	47 (23.6, 17.7-29.5)	46 (23.1, 17.3-29.0)	39 (19.6, 14.1-25.1)
Camel	49	7 (14.3, 4.5-24.1)	4 (8.2, 0.5-15.8)	15 (30.6, 17.7-43.5)	3 (6.1, 0.6-12.8)
Donkey	37	18 (48.6, 32.5-64.8)	19 (51.4, 35.2-67.5)	23 (62.2, 46.5-77.8)	15 (40.5, 24.7-56.4)
Total	3733	338 (9.1, 8.1-10.0)	284 (7.6, 6.8-8.5)	377 (10.1, 9.1-11.1)	241 (6.5, 5.7-7.2)

%, Prevalence. 95% CI, 95% Confidence Interval.

**Table 4:** Specific-*T. gondii* IgM-positive animals based on among SAG1, GRA7 and BAG1 proteins.

Animal	No. tested	Both IgM-SAG1 and IgM-GRA7 positive (%, 95% CI)	Both IgM-BAG1 and IgM-GRA7 positive (%, 95% CI)	Both IgM-SAG1 and IgM-BAG1 positive (%, 95% CI)	Among IgM-SAG1, -GRA7 and-BAG1 positive (%, 95% CI)
Tibetan sheep	904	0	0	0	0
Yak	752	0	0	0	0
Cow	496	0	3 (0.6, 0.1-1.3)	2 (0.4, 0.2-1.0)	0
Pig	456	0	0	0	0
Cattle	451	0	0	1 (0.2, 0.2-0.7)	0
Horse	389	275 (70.7, 66.2-75.2)	145 (37.3, 32.5-42.1)	143 (36.8, 32.0-41.6)	130 (33.4, 28.7-38.1)
Chicken	199	71 (35.7, 29.0-42.3)	30 (15.1, 10.1-20.0)	20 (10.1, 5.9-14.2)	11 (5.5, 2.4-8.7)
Camel	49	/	/	/	/
Donkey	37	/	/	/	/
Total	3733	346 (9.3, 8.3-10.2)	178 (4.8, 4.1-5.5)	166 (4.4, 3.8-5.1)	141 (3.8, 3.2-4.4)

%, Prevalence. 95% CI, 95% Confidence Interval./, no tested.

**Table 5:** Animals with seropositive for at least one specific-*T. gondii* antigen in animals.

Animal	No. tested	Positive animals for at least one specific-*T. gondii* antigen (%, 95% CI)	IgG positive animals for at least one specific antigen (%, 95% CI)	IgM positive animals for at least one specific antigen (%, 95% CI)
Tibetan sheep	904	260 (28.8, 25.8-31.7)	245 (27.1, 24.2-30.0)	30 (3.3, 2.2-4.5)
Yak	752	176 (23.4, 20.4-26.4)	137 (18.2, 15.5-21.0)	47 (6.3, 4.5-8.0)
Cow	496	182 (36.7, 32.5-40.9)	102 (20.6, 17.0-24.1)	129 (26.0, 22.1-29.9)
Pig	456	453 (99.3, 98.6-100.1)	453 (99.3, 98.6-100.1)	3 (0.7, 0.1-1.4)
Cattle	451	82 (18.2, 14.6-21.7)	21 (4.7, 2.7-6.6)	64 (14.2, 11.0-17.4)
Horse	389	372 (95.6,93.6-97.7)	6 (1.5, 0.3-2.8)	373 (95.9, 93.9-97.9)
Chicken	199	195 (98.0, 96.0-99.9)	171 (85.9, 81.1-90.8)	179 (89.9, 85.8-94.1)
Camel	49	39 (79.6, 68.3-90.9)	39 (79.6, 68.3-90.9)	/
Donkey	37	37 (100.0, 100.0)	37 (100.0, 100.0)	/
Total	3733	1796 (48.1, 46.5-49.7)	1111 (29.8, 28.3-31.2)	825 (22.1, 20.8-23.4)

%, Prevalence. 95% CI, 95% Confidence Interval./, no tested.

### 3.4 Analysis of the effect of altitude on the animal seropositive for *T. gondii-*specific antibodies

To determine the influence of altitude on the positivity of *T. gondii* IgG and IgM antibodies, all animals were differentiated into three groups, including 2000-3000, 3000-4000, and 4000-5000 m altitudes (**Table S4**). The results revealed that significant differences (*P* < 0.05) were found in Tibetan sheep, cattle, yaks and horses from different altitudes (**Table S4**).

## 4 Discussion

In this study, serological ELISA diagnostic methods based on *Tg*SAG1, *Tg*GRA7 and *Tg*BAG1 antigens present in the different *T. gondii* forms were established to analyze the positivity rates of specific *T. gondii* IgG and IgM antibodies in Tibetan sheep, yaks, pigs, cows, cattle, horses, chickens, camels and donkeys from the Qinghai-Tibetan Plateau. The differences in IgG or IgM antibody levels in the serum of the same animal were detected among rSAG1-ELISA, rGRA7-ELISA and rBAG1-ELISA. Moreover, there were also large differences in the overall positivity rates of antibodies detected by the three antigens in 3733 serum samples. Therefore, we infer that the parasite forms in which current animal species in the QTPA were infected with *T. gondii*, and the period of infection or the clinical manifestations of the current infection may be different.

Usually, the animals are infected with *T. gondii* by accidentally ingesting oocysts and tissue cysts while eating or drinking (Dubey, 2009; Cenci-Goga et al., 2011; Hill and Dubey, 2013). Sporozoites and bradyzoites were released after the ingestion of oocysts or tissue cysts, invaded the intestinal cells, and quickly differentiated into tachyzoites in 12 and 18 hours, respectively (Dubey, 1997; Dubey et al., 1997). When subjected to strong immune resistance, tachyzoites will transform into bradyzoites to form tissue cysts that persist in the body for a long time. Therefore, throughout the transition process, the bodies of humans and animals will produce specific antibodies against the antigens of the different forms of *T. gondii*. Current specific *T. gondii* antigens used, including SAG1 expressed in the tachyzoite form, GRA7 expressed in sporozoite, tachyzoite and bradyzoite forms, and BAG1 expressed in the bradyzoite form, should be markers for the different parasite stages.

During the natural course of infections, sporozoite- and bradyzoite-specific immune responses are the markers for the diagnosis of cases of firstly acute toxoplasmosis (Döşkaya et al., 2014). Tachyzoite presence indicates that the animal is suffering from acute *T. gondii* infection (Lyons et al., 2002). Moreover, during *T. gondii* infections, the positivity for IgM antibody is proposed to be a marker of acute infection due to the occurrence of IgM antibody within days to a couple of weeks, and IgG antibodies are often interpreted as rising to protective levels after infection and remaining detectable for years, while the positivity for both IgG and IgM antibodies resulted is generally considered to indicate chronic reactivated cases (Kimbita et al., 2001; Döşkaya et al., 2014; Dhakal et al., 2015). IgM antibodies may be detected in humans or animals for a long time following primary *T. gondii* infections (Del Bono et al., 1989; Fricker-Hidalgo et al., 2013; Dhakal et al., 2015; Ybanez et al., 2020; Teimouri et al., 2021), and the natural IgM antibody may interact with parasite antigens in the absence of the *Toxoplasma* infection (Potasman et al., 1986; Sensini et al., 1996.; Liesenfeld et al., 1997; Dhakal et al., 2015; Teimouri et al., 2021). In the current study, the application of specific antigens from different *T. gondii* forms may serve as reliable laboratory tools to confirm the doubts raised by these traditional methods. For the diagnosis of toxoplasmosis in this study, the *Tg*SAG1, *Tg*GRA7, and *Tg*BAG1 antigens from the different parasite forms were used to further analyze both IgG and IgM antibodies. Therefore, these present positive results may represent: the IgG antibody test, rSAG1 + rGRA7 positivity indicates that animals have been infected or are infected currently by tachyzoites, rBAG1 + rGRA7 positivity indicates that animals have been infected by bradyzoites or present tissue cysts, and rSAG1 + rGRA7 + rBAG1 indicates that animals have been infected by parasites or are chronic infections of *T. gondii* currently. The rSAG1 + rGRA7 positivity in IgM antibody tests indicates that the animal is suffering from acute toxoplasmosis caused by tachyzoites; rBAG1 + rGRA7 positivity indicates that the animal is being infected and is in the acute stage of infection caused by bradyzoites; rSAG1 + rGRA7 + rBAG1 indicates that the animal is suffering from acute toxoplasmosis caused by sporozoites, tachyzoites or bradyzoites.

In this work, although it is shown that Tibetan sheep, yaks, cows, and cattle were overall low both for *T. gondii* IgG and IgM positivity, there was no significant difference among rSAG1-, rGRA7-, and rBAG1-ELISAs. These results indicate that *T. gondii* may be infected at low titers in the animals collected in this study. However, the positive rates of 4.1% in yaks, 23.8% in cows and 8.0% in cattle in rBAG1-IgM were found, suggesting the possibility of elevated IgM antibody levels due to vertical infection or sexual transmission in these animal populations cannot be ruled out (Montoya and Remington, 2008; Lopes et al., 2013; Paquet et al., 2013; Beder and Esenkaya Taşbent, 2020).

Interestingly, three current ELISAs were used to test the serum samples of camels, horses and donkeys. It was found that all ELISAs revealed more than 20.4% IgG positivity in camels and 64.9% IgG positivity in donkeys. However, the positivity rates of the three IgG tests in horses were almost zero, but the positivity rates of IgM were 77.9% for rSAG1-, 86.8% for rGRA7- and 41.9% for rBAG1-ELISA. Moreover, in horse testing, 70.7%, 37.3%, 36.8% and 33.4% high IgM positivity was found in the rSAG1 + rGRA7-, rBAG1 + rGRA7-, rSAG1 + rBAG1- and rSAG1 + rGRA7 + rBAG1-ELISAs, respectively. Therefore, horses in the current study may be suffering from sporozoites, tachyzoites or bradyzoite reactivated-caused acute *Toxoplasma* infection, while camels and donkeys may have experienced acute toxoplasmosis or become long-term carriers of tissue cysts. At the same time, such a high positivity rate of IgG or IgM also indicates that the grass or water for horses, donkeys and camels in the sampling area has been polluted by oocysts excluded by wild or stray cats (Tenter et al., 2000; Cenci-Goga et al., 2011; Robert-Gangneux and Dardé, 2012; Saadatnia and Golkar, 2012; Halonen and Weiss, 2013; Liu et al., 2015; Lourido, 2019; Matta et al., 2021).

Furthermore, the rSAG1-ELISA, rGRA7-ELISA and rBAG1-ELISA tests showed high IgG positivity in current pigs, but for IgM antibody, only three pigs presented IgM positivity (two pigs for IgM-GRA7- and one pig for IgM-BAG1-ELISA), suggesting that current pigs may become long-term carriers of tissue cysts of *T. gondii*. Moreover, the seropositive and seronegative samples based on the current SAG1-IgG in pigs were selected to perform the comparative analysis with the commercial ELISA kits based on *Tg*SAG1 protein. The results revealed an 82% match rate with the commercial kits, and a sensitivity of 89.7% and a specificity of 75.4% of the serological *T. gondii* IgG-ELISA assay based on the SAG1 protein established in pigs in this study. These provide confirmations for currently establishing IgG-ELISA and IgM-ELISA based on the proteins that could apply for the serodiagnosis of *T. gondii* infections.

However, it was found that the results were different from those of other animal species in the toxoplasmosis detection of chickens, that is, the positivity rates of IgG and IgM were very high in all tests. This result demonstrates the fact that acute toxoplasmosis occurred in chickens, as well as the soil, water and food (containing animal meats with oocysts) in the chicken’s surroundings are contaminated with oocysts. Moreover, the chicken’s habit of pecking the soil may lead to greater exposure to the threat of oocysts (Wang et al., 2015). Certainly, in areas with the prevalence of toxoplasmosis, the definitive-host cats, especially stray cats, play a key role in frequently exposing other animals to the infectious source and causing *T. gondii* infections in the Qinghai-Tibetan Plateau (Xia et al., 2022; Yang et al., 2022).

In conclusion, the current study confirmed that the application of *Tg*SAG1, *Tg*GRA7, and *Tg*BAG1 recombinant antigens could successfully be used in the detection of specific IgG and IgM antibodies for the serodiagnosis of *T. gondii* infections in Tibetan sheep, yaks, pigs, cows, cattle, horses, chickens, camels and donkeys from the Qinghai-Tibetan Plateau. However, the limitations of the present study include the lack of application of specific antigens of sporozoites and testing of IgG avidity. Future studies need to develop serodetection tests for IgG and IgM against sporozoite antigens and should also test definitive host cats that live around these animals in this plateau area.

## Data availability statement

The original contributions presented in the study are included in the article/**Supplementary Material**, further inquiries can be directed to the corresponding authors.

## Ethics statement

The animal study was reviewed and approved by Ethics Committee of Qinghai University (License number: SL-2021061). Written informed consent was obtained from the owners for the participation of their animals in this study.

## Author contributions

TQ: Collection of animal samples, Data curation, Formal analysis, Investigation, Writing-review and editing. JA: Investigation. YS: Writing-review and editing. HM: Investigation. MK: Writing-review and editing. XY: Collection of animal samples. JL: Conceptualization, Funding acquisition, Resources, Writing-original draft, Writing-review and editing. All authors contributed to the article and approved the submitted version.

## Supplementary material

Supplementary Figure 1 The geographical location and quantity of sampling in various animals in Qinghai province for serodiagnosis of toxoplasmosis based on *T. gondii*-specific SAG1, GRA7 and BAG1 proteins. The graph shows the average altitudes of sampling sites in the 5 states and 2 cities from which the samples were collected and the number of samples of the different animals in each region. HX, Haixi Mongol and Tibetan Autonomous Prefecture. YS, Yushu Tibetan Autonomous Prefecture. HB, Haibei Tibetan Autonomous Prefecture. HN, Hainan Tibetan Autonomous Prefecture. HUN, Huangnan Tibetan Autonomous Prefecture. GL: Guoluo Tibetan Autonomous Prefecture. XN, Xining city. HD, Haidong city.

Supplementary Figure 2 Amino acid sequence alignment and phylogenetic analyses for *Tg*SAG1, *Tg*GRA7, and *Tg*BAG1 with the related cycle-forming organizations. The sequences included *T. gondii* SAG1 (AFO54849.1), *N. caninum* SAG1 (AAD25091.1), *Sarcocystis neurona* SAG1 (AAK40366.1), *T. gondii* GRA7 (ABE69193.1), *N. caninum* GRA7 (AFB77190.1), *Besnoitia besnoiti* GRA7 (XP_029218567.1), *T. gondii* BAG1 (XP_002365116.1), *N. caninum* BAG1 (BAI44436.1), and *B. besnoiti* BAG1 (XP_029221932.1).

Supplementary Figure 3 The specificity of the current ELISA tests was established in this study. IgG-ELISAs **(A)** and IgM-ELISAs **(B)** based on *Tg*GRA7, *Tg*SAG1, *Tg*BAG1, and GST proteins were performed using mouse anti-*T. gondii*, *N. caninum*, and *S. gigantea* positive sera. At least 5 positive sera were used. The coating concentration of the proteins was 1 μg/mL, and the serum dilutions were 1:100. The results showed that GRA7-, SAG1-, and BAG1-ELISAs only reacted with the positive sera of *T. gondii* but did not respond to the positive sera of *N. caninum* and *S. gigantea*. The GST protein did not react with any serum. *Tg*, *T. gondii*. *Nc*, *N. caninum*. *Sg*, *S. gigantea*.

Supplementary Figure 4 The sensitivity assay of the protein concentration under a positive serum dilution of 1:100. In this study, the coated *Tg*GRA7, *Tg*SAG1, *Tg*BAG1, and GST proteins were diluted 2, 1, 0.5, 0.25, 0.125, and 0.0625 μg/mL, and IgG-ELISAs and IgM-ELISAs were developed at a positive serum dilution of 1:100, 200, 400, 800, 1600 and 3200. The 5 positive mouse sera were used. The results show that the reactions could also occur when the concentration of coated proteins was very low under a positive serum dilution of 1:100. P, positive sera. N, negative sera.

Supplementary Figure 5 The sensitivity assay for the serum dilution. Here, mouse anti-*T. gondii* positive and negative sera were diluted with 1:100, 200, 400, 800, 1600, 3200, 6400, 12800 and 25600 for IgG-ELISAs and IgM-ELISAs based on 1 μg/mL *Tg*GRA7, *Tg*SAG1, *Tg*BAG1, and GST proteins. The 5 positive or negative mouse sera were used. The results showed that the OD values at the serum dilution 1:800-6400 were still greater than the cut-off values (calculated using the OD values of negative controls), while GST protein did not show any response.
