# Supplementary material for: Application of Toxoplasma gondii-specific SAG1, GRA7 and BAG1 proteins in serodiagnosis of animal toxoplasmosis
Source: Front Cell Infect Microbiol. 2022 Dec 15;12:1029768. doi: 10.3389/fcimb.2022.1029768 (PMC9798413; doi:10.3389/fcimb.2022.1029768)
Supplement: Supplementary file 1 [file Table_1.docx]

Table S1. Sampling sites in the study

| Prefecture | Sampling area (altitude) | Yak | Cow | Cattle | Tibetan Sheep | Pig | Chicken | Horse | Camel | Donkey | Total |
| --- | --- | --- | --- | --- | --- | --- | --- | --- | --- | --- | --- |
| Haibei | Menyuan (2866 m) | 20 | 0 | 11 | 36 | 0 | 0 | 0 | 0 | 0 | 67 |
|  | Gangcha (3827 m) | 0 | 0 | 0 | 404 | 0 | 0 | 14 | 0 | 0 | 418 |
|  | Haiyan (3000 m) | 104 | 0 | 0 | 145 | 0 | 0 | 10 | 0 | 0 | 259 |
|  | Total | 124 | 0 | 11 | 585 | 0 | 0 | 24 | 0 | 0 | 744 |
| Hainan | Gonghe (3200 m) | 0 | 0 | 0 | 190 | 0 | 0 | 265 | 0 | 0 | 455 |
|  | Guide (2200 m) | 0 | 0 | 0 | 0 | 30 | 0 | 0 | 0 | 0 | 30 |
|  | Total | 0 | 0 | 0 | 190 | 30 | 0 | 265 | 0 | 0 | 485 |
| Haixi | Delingha (2980 m) | 0 | 0 | 45 | 0 | 30 | 0 | 0 | 0 | 0 | 75 |
|  | Golmud (2780 m) | 20 | 0 | 0 | 0 | 20 | 0 | 0 | 0 | 0 | 40 |
|  | Tianjun (3993 m) | 0 | 0 | 0 | 8 | 0 | 0 | 0 | 0 | 0 | 8 |
|  | Wulan (4000 m) | 0 | 0 | 0 | 0 | 0 | 0 | 0 | 49 | 0 | 49 |
|  | Total | 20 | 0 | 45 | 8 | 50 | 0 | 0 | 49 | 0 | 172 |
| Guoluo | Maqin (4100 m) | 110 | 0 | 50 | 0 | 0 | 0 | 0 | 0 | 0 | 160 |
|  | Darlag (4271 m) | 86 | 0 | 0 | 0 | 0 | 0 | 0 | 0 | 0 | 86 |
|  | Banma (3970 m) | 133 | 0 | 0 | 0 | 0 | 0 | 0 | 0 | 0 | 133 |
|  | Total | 329 | 0 | 50 | 0 | 0 | 0 | 0 | 0 | 0 | 379 |
| Huangnan | Jianzha (2063 m) | 0 | 0 | 45 | 45 | 0 | 0 | 0 | 0 | 0 | 90 |
|  | Henan (4000 m) | 0 | 0 | 0 | 0 | 0 | 0 | 60 | 0 | 0 | 60 |
|  | Total | 0 | 0 | 45 | 45 | 0 | 0 | 60 | 0 | 0 | 150 |
| Haidong | Huzhu (2535 m) | 40 | 0 | 0 | 21 | 48 | 30 | 0 | 0 | 37 | 176 |
|  | Ledu (2000 m) | 20 | 289 | 100 | 0 | 67 | 0 | 0 | 0 | 0 | 476 |
|  | Minhe (2174 m) | 0 | 207 | 0 | 0 | 0 | 19 | 0 | 0 | 0 | 226 |
|  | Pingan (2183 m) | 0 | 0 | 0 | 55 | 77 | 0 | 0 | 0 | 0 | 132 |
|  | Total | 60 | 496 | 100 | 76 | 192 | 49 | 0 | 0 | 37 | 1010 |
| Xining | Datong (2756 m) | 219 | 0 | 200 | 0 | 24 | 80 | 30 | 0 | 0 | 553 |
|  | Huangzhong (2645 m) | 0 | 0 | 0 | 0 | 68 | 40 | 10 | 0 | 0 | 118 |
|  | Huangyuan (2660 m) | 0 | 0 | 0 | 0 | 48 | 30 | 0 | 0 | 0 | 78 |
|  | Xining (2261 m) | 0 | 0 | 0 | 0 | 44 | 0 | 0 | 0 | 0 | 44 |
|  | Total | 219 | 0 | 200 | 0 | 184 | 150 | 40 | 0 | 0 | 793 |
| Total |  | 752 | 496 | 451 | 904 | 456 | 199 | 389 | 49 | 37 | 3733 |
