# Supplementary material for: Application of Toxoplasma gondii-specific SAG1, GRA7 and BAG1 proteins in serodiagnosis of animal toxoplasmosis
Source: Front Cell Infect Microbiol. 2022 Dec 15;12:1029768. doi: 10.3389/fcimb.2022.1029768 (PMC9798413; doi:10.3389/fcimb.2022.1029768)
Supplement: Supplementary file 2 [file Table_2.docx]

Table S2 Secondary antibodies used in this study

| Antibodies | Catalog #, company, country |
| --- | --- |
| Goat Anti-Mouse IgG H&L/HRP | G21040, Invitrogen, USA |
| Goat Anti-Mouse IgM H&L/HRP | bs-0368G, Bioss, China |
| Rabbit Anti-Sheep IgM/HRP | ab112763, abcam, UK |
| Rabbit Anti-Sheep IgG H&L/HRP | AS023, Abclonal, China |
| Rabbit Anti-Pig IgG/HRP | bs-0309R-HRP, Bioss, China |
| HRP*Mab Pig IgM | Primadiagnostic, China |
| Rabbit Anti-Bovine IgG H&L/HRP | bs-0326R-HRP, Bioss, China |
| Rabbit Anti-Bovine IgM/HRP | bs-0327R-HRP, Bioss, China |
| Goat Anti-Horse IgM H&L/HRP | ab112879, abcam, UK |
| Rabbit Anti-Horse IgG/HRP | bs-0308R-HRP, Bioss, China |
| Goat Anti-Cow IgG H&L/HRP | ab102154, abcam, UK |
| Sheep Anti-Cow IgM H&L/HRP | ab112752, abcam, UK |
| Goat Anti-Chicken IgG/HRP | bs-0310G-HRP, Bioss, China |
| Rabbit Anti-Chicken IgM/HRP | bs-0314R-HRP, Bioss, China |
| Goat Anti-Donkey IgG H&L/HRP | ab6988, abcam, UK |
| Goat Anti-Camel IgG H and L/HRP | S003H, Nbbiolab, China |
