# Supplementary material for: Application of Toxoplasma gondii-specific SAG1, GRA7 and BAG1 proteins in serodiagnosis of animal toxoplasmosis
Source: Front Cell Infect Microbiol. 2022 Dec 15;12:1029768. doi: 10.3389/fcimb.2022.1029768 (PMC9798413; doi:10.3389/fcimb.2022.1029768)
Supplement: Supplementary file 3 [file Table_3.docx]

Table S3 Comparative analysis of seropositive for *T. gondii* IgG in pig samples between the commercial ELISA kit based on SAG1 and the current ELISAs

| Number | Commercial ELISA kit | rSAG1-ELISA | rGRA7-ELISA | rBAG1-ELISA |
| --- | --- | --- | --- | --- |
| 1 | + | + | + | + |
| 2 | + | + | + | + |
| 3 | + | + | + | + |
| 4 | + | + | + | + |
| 5 | + | + | + | + |
| 6 | + | + | + | + |
| 7 | + | + | + | + |
| 8 | + | + | + | + |
| 9 | + | + | + | + |
| 10 | + | + | + | + |
| 11 | + | + | + | + |
| 12 | + | + | + | + |
| 13 | + | + | + | + |
| 14 | + | + | + | + |
| 15 | + | + | + | - |
| 16 | + | + | + | + |
| 17 | + | + | + | + |
| 18 | + | + | + | + |
| 19 | + | + | + | + |
| 20 | + | + | + | + |
| 21 | + | + | + | + |
| 22 | + | + | + | + |
| 23 | + | + | + | + |
| 24 | + | + | + | + |
| 25 | + | + | + | + |
| 26 | + | + | + | + |
| 27 | + | + | + | - |
| 28 | + | + | + | + |
| 29 | + | + | + | + |
| 30 | + | + | + | + |
| 31 | + | + | + | + |
| 32 | + | + | + | - |
| 33 | + | + | + | + |
| 34 | + | + | - | + |
| 35 | + | + | + | + |
| 36 | + | - | - | - |
| 37 | + | - | + | + |
| 38 | + | - | + | + |
| 39 | + | - | + | + |
| 40 | - | + | + | - |
| 41 | - | + | + | + |
| 42 | - | + | + | - |
| 43 | - | + | + | + |
| 44 | - | + | + | + |
| 45 | - | + | + | + |
| 46 | - | + | + | + |
| 47 | - | + | + | + |
| 48 | - | + | + | + |
| 49 | - | + | + | + |
| 50 | - | + | + | + |
| 51 | - | + | + | - |
| 52 | - | + | + | + |
| 53 | - | + | + | - |
| 54 | - | + | + | + |
| 55 | - | - | - | + |
| 56 | - | - | - | + |
| 57 | - | - | - | - |
| 58 | - | - | - | + |
| 59 | - | - | - | + |
| 60 | - | - | - | + |
| 61 | - | - | + | + |
| 62 | - | - | + | + |
| 63 | - | - | - | + |
| 64 | - | - | + | - |
| 65 | - | - | + | + |
| 66 | - | - | + | + |
| 67 | - | - | - | + |
| 68 | - | - | - | - |
| 69 | - | - | - | + |
| 70 | - | - | - | + |
| 71 | - | - | - | - |
| 72 | - | - | - | + |
| 73 | - | - | - | + |
| 74 | - | - | - | - |
| 75 | - | - | - | - |
| 76 | - | - | + | + |
| 77 | - | - | + | - |
| 78 | - | - | - | + |
| 79 | - | - | - | - |
| 80 | - | - | + | + |
| 81 | - | - | + | + |
| 82 | - | - | + | - |
| 83 | - | - | - | - |
| 84 | - | - | - | + |
| 85 | - | - | - | - |
| 86 | - | - | - | + |
| 87 | - | - | - | - |
| 88 | - | - | + | - |
| 89 | - | - | - | + |
| 90 | - | - | - | - |
| 91 | - | - | - | + |
| 92 | - | - | - | - |
| 93 | - | - | + | - |
| 94 | - | - | - | + |
| 95 | - | - | - | - |
| 96 | - | - | - | + |
| 97 | - | - | - | - |
| 98 | - | - | - | + |
| 99 | - | - | - | + |
| 100 | - | - | + | + |

+, seropositive. -, seronegative.
