# Supplementary material for: Application of Toxoplasma gondii-specific SAG1, GRA7 and BAG1 proteins in serodiagnosis of animal toxoplasmosis
Source: Front Cell Infect Microbiol. 2022 Dec 15;12:1029768. doi: 10.3389/fcimb.2022.1029768 (PMC9798413; doi:10.3389/fcimb.2022.1029768)
Supplement: Supplementary file 4 [file Table_4.docx]

Table S4 Analysis of influence of altitude on seropositivity of *T. gondii* IgG and IgM antibodies

| ELISA | Animal | 2000-3000m | | 3000m-4000m | | 4000m-5000m | | *P*-value |
| --- | --- | --- | --- | --- | --- | --- | --- | --- |
|  |  | Tested | Positive (%, 95% CI) | Tested | Positive (%, 95% CI) | Tested | Positive (%, 95% CI) |  |
| SAG1-IgG | Tibetan sheep | 157 | 53 (33.8, 26.4-41.2) | 747 | 110 (14.7, 12.2-17.3) | 0 | - | <0.0001 |
|  | Yak | 299 | 7 (2.3, 0.6-4.1) | 237 | 1 (0.4, 0.4-1.2) | 216 | 8 (3.7, 1.2-6.2) | 0.0572 |
|  | Cow | 496 | 1 (0.2, 0.2-0.6) | 0 | - | 0 | - | - |
|  | Pig | 456 | 397 (87.1, 84.0-90.1) | 0 | - | 0 | - | - |
|  | Cattle | 401 | 10 (2.5, 1.0-4.0) | 0 | - | 50 | 0 | - |
|  | Horse | 40 | 0 | 289 | 0 | 60 | 0 | - |
|  | Chicken | 199 | 139 (69.8, 63.5-76.2) | 0 | - | 0 | - | - |
|  | Camel | 0 | - | 0 | - | 49 | 33 (67.3, 54.2-80.5) | - |
|  | Donkey | 37 | 28 (75.7, 61.9-89.5) | 0 | - | 0 | - | - |
| GRA7-IgG | Tibetan sheep | 157 | 28 (17.8, 11.8-23.8) | 747 | 60 (8.0, 6.1-10.0) | 0 | - | 0.0010 |
|  | Yak | 299 | 6 (2.0, 0.4-3.6) | 237 | 2 (0.8, 0.3-2.0) | 216 | 5 (2.3, 0.3-4.3) | 0.4460 |
|  | Cow | 496 | 1 (0.2, 0.2-0.6) | 0 | - | 0 | - | - |
|  | Pig | 456 | 297 (65.1, 60.8-69.5) | 0 | - | 0 | - | - |
|  | Cattle | 401 | 2 (0.5, 0.2-1.2) | 0 | - | 50 | 5 (10.0, 1.7-18.3) | 0.0001 |
|  | Horse | 40 | 0 | 289 | 2 (0.7, 0.3-1.6) | 60 | 0 | 0.7079 |
|  | Chicken | 199 | 128 (64.3, 57.7-71.0) | 0 | - | 0 | - | - |
|  | Camel | 0 | - | 0 | - | 49 | 10 (20.4, 9.1-31.7) | - |
|  | Donkey | 37 | 24 (64.9, 49.5-80.2) | 0 | - | 0 | - | - |
| BAG1-IgG | Tibetan sheep | 157 | 10 (6.4, 2.5-10.2) | 747 | 27 (3.6, 2.3-5.0) | 0 | - | 0.1317 |
|  | Yak | 299 | 44 (14.7, 10.7-18.7) | 237 | 47 (19.8, 14.8-24.9) | 216 | 22 (10.2, 6.2-14.2) | 0.0471 |
|  | Cow | 496 | 100 (20.2, 16.6-23.7) | 0 | 0 | 0 | - | - |
|  | Pig | 456 | 315 (69.1, 64.8-73.3) | 0 | 0 | 0 | - | - |
|  | Cattle | 401 | 6 (1.5, 0.3-2.7) | 0 | - | 50 | 0 | 0.3875 |
|  | Horse | 40 | 0 | 289 | 4 (1.4, 0.0-2.7) | 60 | 0 | 0.5018 |
|  | Chicken | 199 | 56 (28.1, 21.9-34.4) | 0 | - | 0 | - | - |
|  | Camel | 0 | - | 0 | - | 49 | 19 (38.8, 25.1-52.4) | - |
|  | Donkey | 37 | 30 (81.1, 68.5-93.7) | 0 | - | 0 | - | - |
| SAG1-IgM | Tibetan sheep | 157 | 7 (4.5, 1.2-7.7) | 747 | 6 (0.8, 0.2-1.4) | 0 | - | 0.4366 |
|  | Yak | 299 | 4 (1.3, 0.0-2.6) | 237 | 2 (0.8, 0.3-2.0) | 216 | 7 (3.2, 0.9-5.6) | 0.1292 |
|  | Cow | 496 | 3 | 0 | - | 0 | - | - |
|  | Pig | 456 | 0 | 0 | - | 0 | - | - |
|  | Cattle | 401 | 10 (2.5, 1.0-4.0) | 0 | - | 50 | 1 (2.0, 1.9-5.9) | 0.0007 |
|  | Horse | 40 | 32 (80.0, 67.6-92.4) | 289 | 215 (74.4, 69.4-79.4) | 60 | 56 (93.3, 87.0-99.6) | 0.5426 |
|  | Chicken | 199 | 96 (48.2, 41.3-55.2) | 0 | - | 0 | - | - |
| GRA7-IgM | Tibetan sheep | 157 | 3 (1.9, 0.2-4.1) | 747 | 3 (0.4, 0.1-0.9) | 0 | - | 0.0364 |
|  | Yak | 299 | 2 (0.7, 0.3-1.6) | 237 | 1 (0.4, 0.4-1.2) | 216 | 1 (0.5, 0.4-1.4) | 0.9150 |
|  | Cow | 496 | 11 (2.2, 0.9-3.5) | 0 | - | 0 | - | - |
|  | Pig | 456 | 2 (0.4, 0.2-1.0) | 0 | - | 0 | - | - |
|  | Cattle | 401 | 17 (4.2, 2.3-6.2) | 0 | - | 50 | 1 (2.0, 1.9-5.9) | 0.4598 |
|  | Horse | 40 | 36 (90.0, 80.7-99.3) | 289 | 243 (84.1,79.9-88.3) | 60 | 58 (96.7, 92.1-101.2) | 0.7788 |
|  | Chicken | 199 | 108 (54.3, 47.3-61.2) | 0 | - | 0 | - | - |
| BAG1-IgM | Tibetan sheep | 157 | 2 (1.3, 0.5-3.0) | 747 | 9 (1.2, 0.4-2.0) | 0 | - | 0.9435 |
|  | Yak | 299 | 11 (3.7, 1.5-5.8) | 237 | 4 (1.7, 0.0-3.3) | 216 | 16 (7.4, 3.9-10.9) | 0.0126 |
|  | Cow | 496 | 118 (23.8, 20.0-27.5) | 0 | - | 0 | - | - |
|  | Pig | 456 | 1 (0.2, 0.2-0.6) | 0 | - | 0 | - | - |
|  | Cattle | 401 | 34 (8.5, 5.8-11.2) | 0 | - | 50 | 2 (4.0, 1.4-9.4) | 0.3011 |
|  | Horse | 40 | 3 (7.5, 0.7-15.7) | 289 | 126 (43.6, 37.9-49.3) | 60 | 34 (56.7, 44.1-69.2) | 0.0018 |
|  | Chicken | 199 | 82 (41.2, 34.4-48.0) | 0 | - | 0 | - | - |

-, no analysis.
