# Supplementary figures and images for: Application of Toxoplasma gondii-specific SAG1, GRA7 and BAG1 proteins in serodiagnosis of animal toxoplasmosis
Source: Front Cell Infect Microbiol. 2022 Dec 15;12:1029768. doi: 10.3389/fcimb.2022.1029768 (PMC9798413; doi:10.3389/fcimb.2022.1029768)

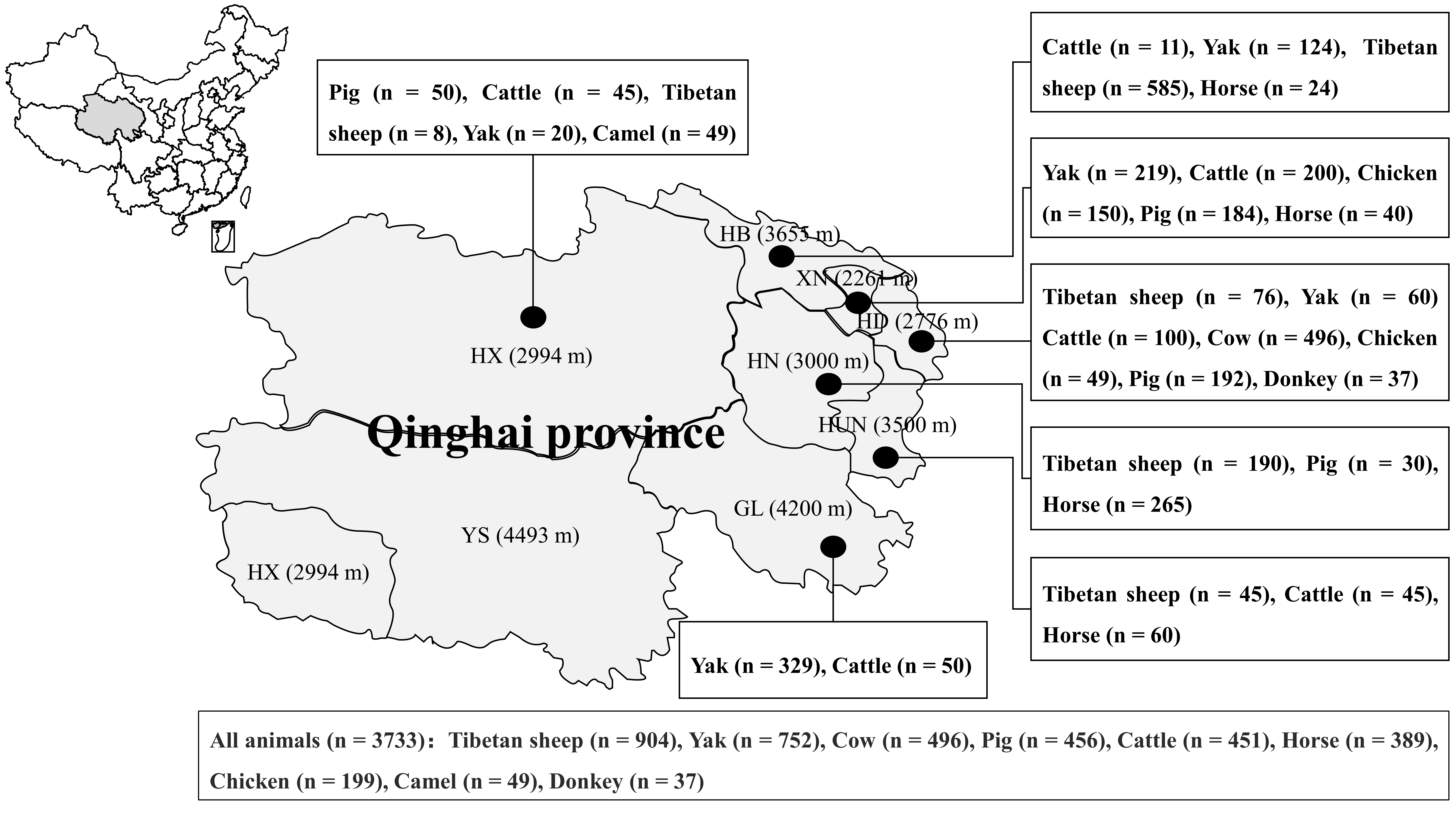

Supplement: Supplementary Figure 1 — The geographical location and quantity of sampling in various animals in Qinghai province for serodiagnosis of toxoplasmosis based on T. gondii-specific SAG1, GRA7 and BAG1 proteins. The graph shows the average altitudes of sampling sites in the 5 states and 2 cities from which the samples were collected and the number of samples of the different animals in each region. HX, Haixi Mongol and Tibetan Autonomous Prefecture. YS, Yushu Tibetan Autonomous Prefecture. HB, Haibei Tibetan Autonomous Prefecture. HN, Hainan Tibetan Autonomous Prefecture. HUN, Huangnan Tibetan Autonomous Prefecture. GL: Guoluo Tibetan Autonomous Prefecture. XN, Xining city. HD, Haidong city. [file Image_1.tif]

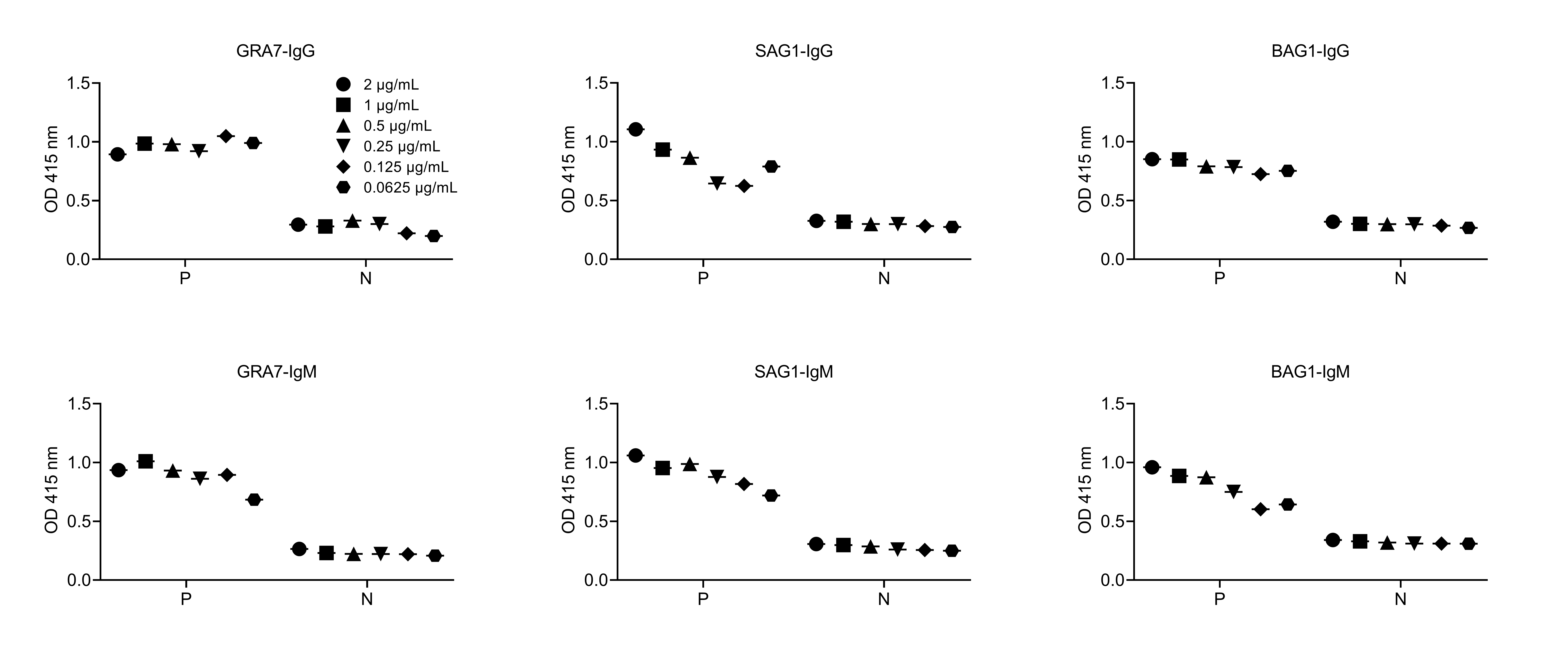

Supplement: Supplementary Figure 4 — The sensitivity assay of the protein concentration under a positive serum dilution of 1:100. In this study, the coated TgGRA7, TgSAG1, TgBAG1, and GST proteins were diluted 2, 1, 0.5, 0.25, 0.125, and 0.0625 μg/mL, and IgG-ELISAs and IgM-ELISAs were developed at a positive serum dilution of 1:100, 200, 400, 800, 1600 and 3200. The 5 positive mouse sera were used. The results show that the reactions could also occur when the concentration of coated proteins was very low under a positive serum dilution of 1:100. P, positive sera. N, negative sera. [file Image_4.tif]

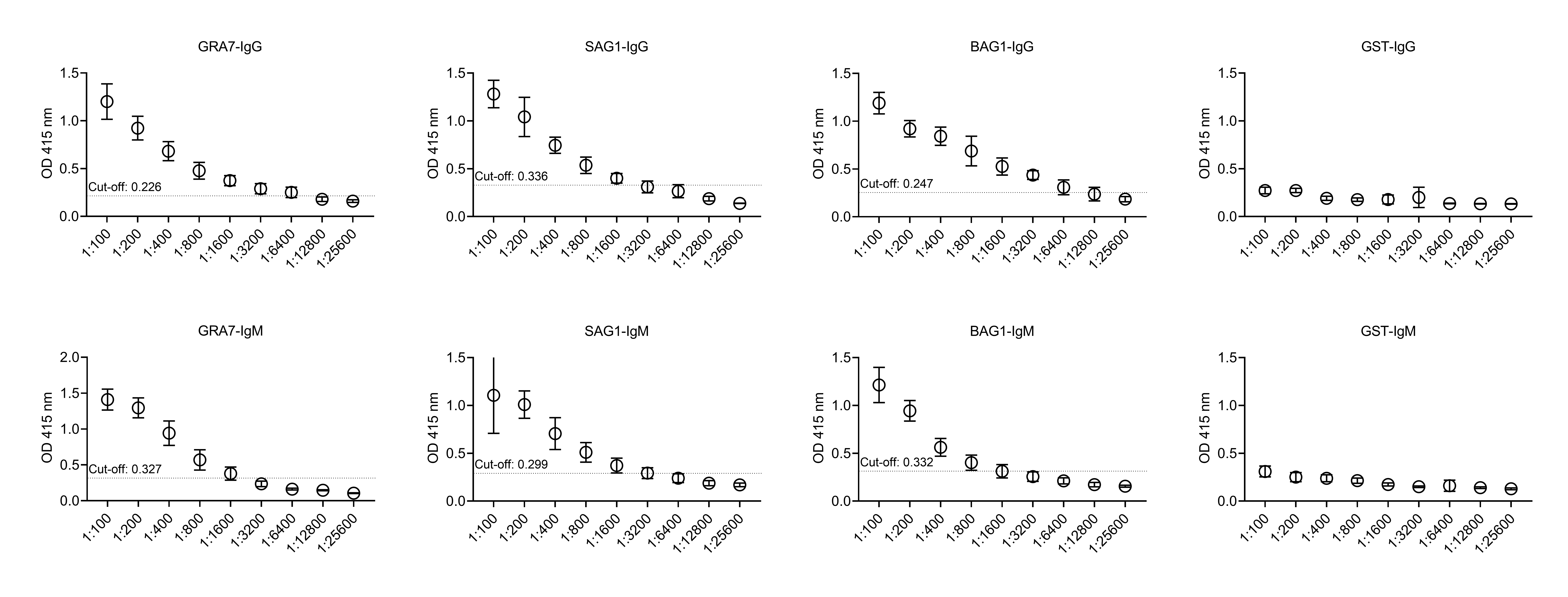

Supplement: Supplementary Figure 5 — The sensitivity assay for the serum dilution. Here, mouse anti-T. gondii positive and negative sera were diluted with 1:100, 200, 400, 800, 1600, 3200, 6400, 12800 and 25600 for IgG-ELISAs and IgM-ELISAs based on 1 μg/mL TgGRA7, TgSAG1, TgBAG1, and GST proteins. The 5 positive or negative mouse sera were used. The results showed that the OD values at the serum dilution 1:800-6400 were still greater than the cut-off values (calculated using the OD values of negative controls), while GST protein did not show any response. [file Image_5.tif]
